# Downregulation of the human peripheral myelin protein 22 gene by miR-29a in cellular models of Charcot–Marie–Tooth disease

**DOI:** 10.1038/s41434-019-0098-z

**Published:** 2019-08-27

**Authors:** Jacquelyn Serfecz, Hannah Bazick, Mohammed Omar Al Salihi, Peter Turner, Christopher Fields, Pedro Cruz, Rolf Renne, Lucia Notterpek

**Affiliations:** 1 Department of Molecular Genetics & Microbiology, College of Medicine University of Florida, Gainesville, FL 32610 USA; 2 Department of Neuroscience, College of Medicine University of Florida, Gainesville, FL 32610 USA; 3 Center for Translational Research in Neurodegenerative Disease, College of Medicine University of Florida, Gainesville, FL 32610 USA; 4 UF Health Cancer Center, College of Medicine University of Florida, Gainesville, FL 32610 USA

**Keywords:** Neurological disorders, Myelin biology and repair

## Abstract

The majority of hereditary neuropathies are caused by duplication of the peripheral myelin protein 22 (PMP22) gene. Therefore, mechanisms to suppress the expression of the PMP22 gene have high therapeutic significance. Here we asked whether the human *PMP22* gene is a target for regulation by microRNA 29a (miR-29a). Using bioinformatics, we determined that the human *PMP22* gene contains the conserved seed sequence of the miR-29a binding site and this regulatory motif is included in the duplicated region in neuropathic patients. Using luciferase reporter assays in HEK293 cells, we demonstrated that transient transfection of a miR-29a mimic is associated with reduction in PMP22 3′UTR reporter activity. Transfecting normal and humanized transgenic neuropathic mouse Schwann cells with a miR-29a expression plasmid effectively lowered both the endogenous mouse and the transgenic human PMP22 transcripts compared with control vector. In dermal fibroblasts derived from neuropathic patients, ectopic expression of miR-29a led to ~50% reduction in PMP22 mRNA, which corresponded to ~20% decrease in PMP22 protein levels. Significantly, miR-29a-mediated reduction in PMP22 mitigated the reduced mitotic capacity of the neuropathic cells. Together, these results support further testing of miR-29a and/or PMP22-targeting siRNAs as therapeutic agents for correcting the aberrant expression of PMP22 in neuropathic patients.

## Introduction

Duplication of the human peripheral myelin protein 22 (PMP22)/growth arrest-specific gene 3 (gas-3), is responsible for Charcot–Marie–Tooth type 1 A (CMT1A) neuropathy, while haploinsufficiency is linked with hereditary neuropathy with liability to pressure palsy (HNPP) [1]. CMT disease is the most common inherited peripheral neuropathy, affecting about 1:2500 individuals [2]. The disease manifests itself as a progressive demyelinating neuropathy, leading to muscle atrophy and numbness due to a dampened peripheral nerve response [1]. CMT1A is caused by a 1.5 Mb tandem duplication of the PMP22 gene on the short arm of chromosome 17 [3, 4]. Although the PMP22 mRNA is ubiquitously expressed throughout most tissues, readily assessable protein expression is restricted to myelinating Schwann cells, suggesting a role for posttranscriptional regulation by microRNAs (miRNAs) [5, 6].

PMP22 comprises 2–5% of the proteins in peripheral nerve myelin and is critical for membrane ultrastructure [7]. Overproduction of PMP22, such as in nerves of CMT1A patients, leads to demyelination and accumulation of misfolded PMP22 [8]. Severity of CMT1A is dependent on PMP22 copy number, as patients with four rather than three copies have a more severe neuropathy [9]. Dose sensitivity of PMP22 is supported by studies in transgenic animals that express either several extra or fewer copies of the PMP22 gene [10]. Therefore, partially reducing PMP22 gene dosage in myelinating Schwann cells via small noncoding RNAs could be an effective treatment for CMT1A patients.

Recently, gene therapy approaches, including antisense oligos (ASOs), have shown efficacy against CMT1A in rodent models [11]. ASOs are synthetic, 20-nucleotide sequences that are completely complementary to the sequence of a disease-associated messenger RNA or microRNA. The duplex RNA that is formed by hybridization of the ASO is then degraded by RNase H, and translation or function is prevented [12]. In comparison with ASOs, miRNAs are endogenously expressed regulatory molecules that are partially mismatched, and incorporated in the RNA-induced silencing complex (RISC) and located to their targets. MiRNAs repress translation and/or induce RNA turnover by targeting the 3′UTR of mRNAs in a reverse complementary manner posttranscriptionally by the RISC [13]. The transcription of miRNAs begins in the nucleus by Pol II, generating a primary microRNA (pri-miRNA) containing a hairpin [14, 15]. Drosha/DGCR, an RNA endonuclease, processes the pri-miRNA at the base of the stem loop in the nucleus to form a precursor microRNA (pre-miRNA) about 70 nts long that gets exported out of the nucleus [16]. While in the cytoplasm, the pre-miRNA gets cleaved by Dicer and one strand preferentially incorporates into the RISC complex which then targets 3′UTR of mRNAs and represses translation [17]. The targeting often depends on the seed sequence at the 5′ end of the miRNA of about six to eight nucleotides in the complementary 3′UTR segment of an mRNA [18].

We previously showed in rodent Schwann cells that PMP22 is regulated by miR-29a, as increased levels of miR-29a suppressed PMP22 transcripts, while inhibition of endogenous miR-29a increased PMP22 expression [6]. In the current study, we asked if targeting human PMP22 with miR-29a would lead to near-normal transcript levels and ameliorate the cellular phenotype of dermal fibroblasts from CMT1A patients [19].

## Materials and methods

### Primary mouse Schwann cell cultures

C57Bl/6J wild-type and PMP22 overexpressor (C22) [20] mouse colonies were housed under SPF conditions at the McKnight Brain Institute animal facility. The use of animals for these studies was approved by the University of Florida Institutional Animal Care and Use Committee (IACUC). Primary mouse Schwann cell cultures were established from the sciatic nerves of postnatal day 3 (P3) to P7 genotyped pups [21].

### Human dermal fibroblast cultures

Human skin fibroblasts from two CMT1A patients (P2: GM05167 and P4: GM05165) were purchased from the Coriell Institute (Camden, New Jersey). Skin fibroblasts from nonneuropathic individuals were obtained under an IRB-approved protocol by Dr Guang-Bin Xia (Department of Neurology, University of Florida) and provided to us for the described studies. Cultures were seeded at 0.1 × 10^6^ cells per mL and maintained in Dulbecco’s Modified Essential Medium (DMEM), supplemented with 10% fetal bovine serum (FBS) [21]. Procedures with the human cells were carried out in compliance with IRB-approved procedures. Fibroblast cultures below eight passages were used for all experiments.

### Transfection with miR29 mimics

Cells (HEK293, human dermal fibroblasts) were maintained in appropriate DMEM supplemented with 10% FBS and 20 mmol/L l-glutamine. The miR-29a mimics and control nonspecific mimics (synthetic double-stranded mature miRNAs) were purchased from Dharmacon (Lafayette, CO). Cultures were transiently transfected with human microRNA miRIDIAN hsa-miR-29a-3p mimic (Cat # C-300504-07) or Dharmacon miRIDIAN microRNA mimic Negative Control (Cat # CN-001000-01-05) (nonspecific mimic). Cells were seeded in 6-well or 24-well plates for 48 h before transfection with nonspecific mimic or miR29 mimics (25 and 50 nM) using Lipofectamine RNAiMAX (Invitrogen, Carlsbad, USA) in serum-free media. After 6 h, the media was changed for transfection efficiency assays and the cultures were maintained in media containing 15% FBS for an additional 24 h. For quantitative RT-PCR, mRNA was harvested 48 h post transfection. For protein analyses, the cell lysates were prepared 72 h post transfection.

### Luciferase assays

HEK293 cells were maintained in DMEM, supplemented with 10% FBS and 1% penicillin–streptomycin. Cells were seeded into 24-well plates at a concentration of 1.5 × 10^5^ per mL. Lipofectamine 3000 (Invitrogen) co-transfection was performed with 20 ng pEZX-MT01-PMP22-3´-UTR reporter plasmid (GeneCopoeia) and either the pTR2 plasmid (50 and 80 ng) constitutively expressing the pre-miRNA or a human microRNA miRIDIAN hsa-miR-29a-3p mimic (2, 5, and 10 nM) (GE Dharmacon). pEZX-MT01 reporter vector contains the firefly luciferase gene immediately upstream of the PMP22 3′-UTR and an independent Renilla luciferase gene as an internal control for normalization. After 48 h of the co-transfection, a dual luciferase assay was performed using the dual-luciferase assay kit (Promega, Madison, Wisconsin) on a FLUOstar Optima microplate reader (BMG Labtech, Cary, NC). The ratio of firefly to Renilla luciferase was measured and normalized relative to the cells transfected with the pEZX-MT01-PMP22-3’-UTR vector and human microRNA miRIDIAN nonspecific mimics.

### Transfection of mouse Schwann cell cultures

Primary mouse Schwann cells from WT and C22 mice were transfected with 1 µg of empty pCMV vector (CAT#: PCMVMIR) or with miR-29a (ORIGENE MI0000087) for 6 h using MegaTran (ORIGENE TT200003). The standard ratio of 3 µl of MegaTran to 1 µg of DNA was used. Subsequently, the media was changed to DMEM-F12 with 15% FBS, 20 µg/ml bovine pituitary extract (Biomedical Technologies Inc.) and 5 µM forskolin (Calbiochem, Burlington, MA) for 24 h. Cultures were harvested in QIAzol (QIAGEN) and total RNA was isolated according to the manufacturer’s instructions.

### Quantitative real-time PCR analyses

Total RNA (700 ng) isolated from the mouse Schwann cell cultures was reverse transcribed using high-capacity RNA-to-cDNA kit (Applied Biosystems). For quantitative RT-PCR, undiluted cDNA and primers specific for mouse and human PMP22 or RPL32 were used with the SYBR Green FastStart PCR Master Mix (Roche, Basel, Switzerland). Mouse PMP22 Forward Primer: 5′-CCG CAG CAC AGC TGT CTT T-3′; Mouse PMP22 Reverse Primer: 5′-AGC AGA TTA GCC TCA GGC ACA A-3′; Human PMP22 Forward Primer: 5′-CTC CTC CTG TTG CTG AGT ATC-3′; Human PMP22 Reverse Primer: 5′-GCT ACA GTT CTG CCA GAG A-3′ [11]. The relative expression of the human and the mouse PMP22 to RPL32 mRNAs were determined using 2^−∆∆CT^ method [22].

Cultures of human dermal fibroblasts were harvested in RNA-Bee (Tel Test). Total RNA (900 ng) was reverse transcribed using high-capacity RNA-to-cDNA kit (Applied Biosystems, Foster City, CA). For quantitative RT-PCR analyses, undiluted cDNA and primers for human PMP22 or GAPDH were used with the SYBR Green FastStart PCR Master Mix (Roche). For the amplification of PMP22, the primers were designed to amplify a 237 bp region within the first coding exon of the human gene, recognizing transcripts containing both exon 1A and exon 1B [23]. PMP22 Forward Primer: 5′ GTA TCA TCG TCC TCC ACG TC 3′; PMP22 Reverse Primer: 5′ GGC AGA AGA ACA GGA ACA GA 3′. The relative expression of PMP22 to GAPDH mRNA was determined using the 2^−ΔΔCT^ method [22].

### Digital fluorescent immunoblotting

Confluent cultures of CMT1A patient fibroblasts were transiently transfected with human microRNA miRIDIAN hsa-miR-29a-3p mimic, or Dharmacon miRIDIAN microRNA nonspecific mimic, or Dharmacon siGLO Red Transfection Indicator using Lipofectamine RNAiMAX (Invitrogen). After 72 h of transfection, the cells were lysed in radioimmunoprecipitation assay buffer, containing 3% SDS and complete protease inhibitors (Roche). Pierce BCA assays were performed on each lysate (Thermo Fisher, Waltham, MA). For carbohydrate modification analysis of PMP22, total cell lysates (10 µg) were treated with N-glycosidase F (PNGase F) (New England Biolabs) per manufacturer instructions. Proteins were separated on 12.5% SDS gels, followed by transfer to PVDF membranes. After blocking in Odyssey blocking buffer (TBS), blots were incubated overnight at 4 °C with rabbit polyclonal anti-PMP22 [24] and goat anti-GAPDH (Santa Cruz, CA). Subsequently, the blots were incubated in a 1:15,000 secondary IRDye donkey anti-rabbit and anti-goat antibodies, and imaged on the CLx system (LiCor, Lincoln, NE). Using Image Studio 5 (LiCor), images with nonsaturated pixels were acquired and used for quantification. The background of the blot was determined by averaging the pixel intensity of the chosen region with no protein signal. The signal of each protein band was determined by measuring the sum of the individual pixel intensities within the selection region containing the band, and then subtracting the background value calculated previously. PMP22 signals were then normalized to each corresponding GAPDH control signals, acquired under identical conditions from the same membrane.

### Plasmids

Plasmids constructed from pTR2-CB-dTomato-3×-Myc-WPRE-MCS were generated with the GeneArt Seamless Cloning and Assembly Kit (Life Technologies, Carlsbad, CA). MiR-29a was engineered using primers that amplify miR-29a pre-miRNA from pCMV-miR-29a plasmid (Origene, Rockville, MD). An siRNA was made using the same primers from a template including a hairpin, which is perfectly complementary to the binding site in the 3′UTR of PMP22 (designed and ordered gBlock from IDT). Plasmids containing a luciferase reporter were constructed as follows: linearized pTR2 was cut with XbaI and XhoI restriction enzymes and a 6 kb fragment was excised from a 1% agarose gel. These cut sites were selected to avoid interference from tdTomato fluorescence. Full miR-29a and siRNA inserts were PCR amplified with the following primers:

Forward: 5′-**CGTGTGACCGGCGGC**ATACTACACCATTTTCTATCA-3′

Reverse: 5′-**ATTATCGATAAGCTG**CCAGGAGTGTTTCTAGGTATCCG-3′. The bolded sequences indicate homology with pTR2. These amplicons were generated using NEB Phusion High Fidelity DNA Polymerase with <18 amplification cycles. DNA was purified with the Monarch PCR cleanup Kit (New England Biolabs, Ipswich, MA), and plasmids assembled by GeneArt Cloning. Each reaction was set up so that 20 ng of amplicon was mixed with 100 ng of pTR2 vector in 5× reaction buffer. The reaction mix was transformed into TOP10 competent cells per the manufacturer’s protocol. Clones were screened via restriction digests and analyzed. Selected colonies were harvested with a Qiaprep Spin Mini Prep Kit (Qiagen, Hilden, Germany).

AAV2 plasmids were constructed as follows: linearized pTR2 was cut with XbaI and HindIII and a 7 kb fragment was purified by agarose gel electrophoresis. Full miR-29a and siRNA inserts were PCR amplified with

Forward: 5′-**CGTGTGACCGGCGGC**ATACTACACCATTTTCTATCA-3′ and

Reverse: 5′-**AGTGATATCCAATTG**CCAGGAGTGTTTCTAGGTATCCG-3′ primers.

The bold sequences indicate homology with pTR2. PCR reactions and GeneArt cloning were performed as above. Clones were screened by restriction digestion and sequenced. MiRNA, siRNA, and miRNA sensor AAV expression plasmids, driven by the CMV enhancer, chicken beta actin promoter, were constructed (pTR2-CB-tdTomato-WPRE-MCS). The AAV plasmid backbone pTR2-CB-dTomato-WPRE-MCS has been described before [25]. TdTomato reporter gene was subcloned from previous AAV plasmid [26]. Integrity of the ITRs was checked routinely by restriction digestion with SmaI. AAV2 virus preparation has been described previously [27]. Integrity of the ITRs was checked routinely by restriction digestion with DraIII and SnaBI (ITR1) and HincII and SapI (ITR2).

### Cell division studies in human dermal fibroblasts

After 48 h of transfection with miR-29a mimics, cultures were stained with Hoechst dye and the number of cells in a fixed field was counted. For infection studies, supernatants of AAV2 viral particles either expressing miR-29a (titer: 2.82e + 13) a hardened siRNA-like mutant of miR-29a (titer: 3.71E + 13) or empty TR2 (titer: 2.15E + 13) vectors were incubated with the dermal fibroblasts for 72 h. Next, the cells were fixed with 4% paraformaldehyde for 10 min, permeabilized with 0.1% Triton X-100 for 10 min at room temperature, rinsed in PBS, and processed for immunolabeling with rabbit anti-Ki67 (EnCor, Gainesville, FL) and mouse antivimentin (Santa Cruz) antibodies overnight at 4 °C. Bound primary antibodies were detected with anti-rabbit and anti-mouse IgG secondary antibodies (Invitrogen). Nuclei were stained with Hoechst dye (Invitrogen) and coverslips were mounted using ProLong Antifade mounting medium (Invitrogen). To determine the mitotic capacity of the cultures, Ki67-positive cells were counted in a fixed field (0.8 mm^2^), and divided by the total number of Hoechst dye positive cells. From each condition, the total number of cells in 6 to 170 randomly chosen fields were counted and graphed.

### Statistical analyses

For all experiments, means ± standard error mean (S.E.M.) were calculated. Significance was determined by performing paired or unpaired, one- or two-tailed Student’s *t*-tests, using GraphPad Prism software. Statistical significance was denoted by a *P* value < 0.05.

## Results

### Human PMP22 is a target of miR-29a

**Fig. 1:**
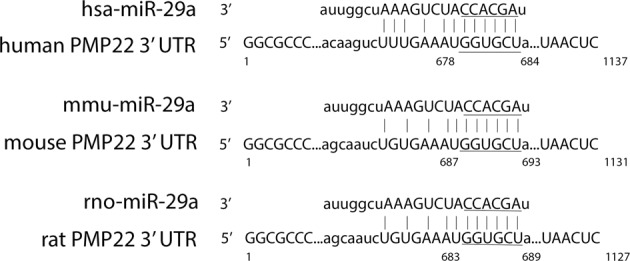
The miR-29a binding site within the PMP22 3′-UTR is conserved between human, mouse, and rat species. Alignments of the miR-29a binding site in the 3′-UTR of the human, mouse, and rat PMP22 transcripts are shown. The seed region for the miR-29a binding site is underlined. Alignment from www.microRNA.org

In previous work, we reported on the regulation of PMP22 by miR-29a in rodent cells, where overexpression of miR-29a reduced steady-state PMP22 levels, and inhibition of endogenous miR-29a relieved the miRNA-mediated repression [6]. Bioinformatics predictions similarly identified a miR-29a target site within the 3′-UTR of the human PMP22 gene (Fig. 1). The 3′-UTR of the human PMP22 mRNA is ~1.1 kb and hsa-miR-29a targets a region about 300 bp upstream from the PolyA signal. As shown on the alignments, both miR-29a and its target sites within the 3′-UTR of PMP22 are conserved between human, mouse, and rat species (Fig. 1).

**Fig. 2:**
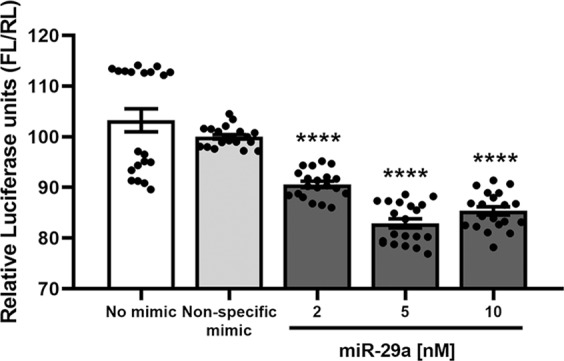
Reporter assays validate the regulation of the human PMP22 3′UTR by miR-29a. Firefly/Renilla (FL/RL) dual luciferase assays were performed in HEK293 cells after transient transfection with nonspecific mimic control or 2, 5, or 10 nM miR-29a mimic. An ~20% decrease in relative luciferase activity is observed when 20 ng of reporter vector pEZX-MT01 was co-transfected with miR-29a, but not with nonspecific mimic. Data points from *N* = 2 independent cultures per sample, with ten technical replicates of each condition, are shown. *P*-values < 0.0001 (****) were considered significant, compared with both no mimic and nonspecific mimic conditions, using unpaired one-tailed Student’s *t*-tests

To test the functionality of miR-29a in regulating the human PMP22 transcript, we used Firefly/Renilla (FL/RL) dual luciferase assays (Fig. 2). This approach utilizes a 3′-UTR reporter to identify gene targets undergoing microRNA-mediated post transcriptional regulation. Upon transient transfection of HEK293 cells with miR-29a mimics, a moderate but significant decline of the human 3′-UTR PMP22 reporter-driven luciferase signal is observed (Fig. 2). The miR-29a mimics are similarly effective at 2, 5, and 10 nM concentrations, while the nonspecific mimic has no influence on the reporter signal. These data demonstrate that similar to the rodent gene, the human PMP22 3′-UTR is a target of miR-29a.

### MiR-29a regulates PMP22 in Schwann cells from neuropathic mice

**Fig. 3:**
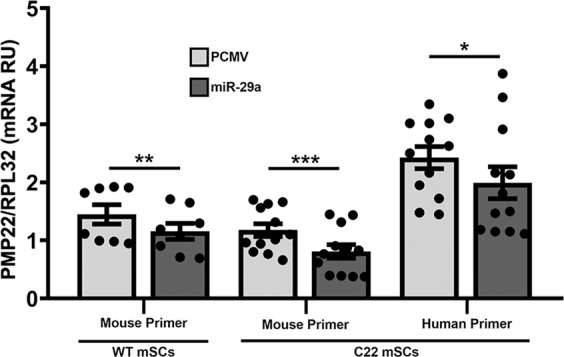
Suppression of the mouse and the human PMP22 transcripts in primary cultured mouse Schwann cells from WT and C22 neuropathic mice by miR-29a. Schwann cells from WT and C22 mice were transiently transfected with empty pCMV or with the pCMV-miR-29a and analyzed for the mouse and human PMP22 mRNAs. RT-PCR with specific primers to the mouse and the human PMP22 transcripts show the inhibition of the endogenous mouse gene, and the suppression of the exogenous human transgene in the neuropathic C22 cells. *p < 0.05, **p < 0.01, ***p < 0.001 paired one-tailed Student’s *t*-tests. Individual data points are graphed from *N* = 2–3 independent cultures per samples, with four technical replicates each. RU, Relative Units

C22 mice, a transgenic rodent model of CMT1A, have integrated seven copies of the human *PMP22* gene and express ~1.7 times more human PMP22 mRNA than the endogenous mouse locus [20]. Schwann cells from C22 mice display features of the human disease, including impaired capacity for myelin expansion [28]. To test the efficacy of miR-29a in regulating the human PMP22 gene in Schwann cells, we transiently transfected cells from WT and C22 neuropathic mice with empty pCMV or pCMV-miR-29a expression plasmids (Fig. 3). Transfection of WT mouse Schwann cells with miR-29a yields an ~25% reduction in the endogenous mouse transcript, as compared with empty vector. In neuropathic C22 Schwann cells transfected with the control vector, we detect elevated levels of the human PMP22, as compared with the endogenous mouse transcript. As expected, in cells from C22 mice, expression of both the endogenous mouse PMP22 gene and the exogenous human PMP22 transgene are reduced upon transfection with the miR-29a plasmid. Quantification from independent experiments indicate the reduction of the human PMP22 mRNA by ~16% (Fig. 3). The modest reduction in the levels of the human PMP22 mRNA is likely a reflection of the low transfection efficiency of mouse Schwann cells, which is only ~15–20%.

### Suppression of human PMP22 in dermal fibroblasts from CMT1A patients

**Fig. 4:**
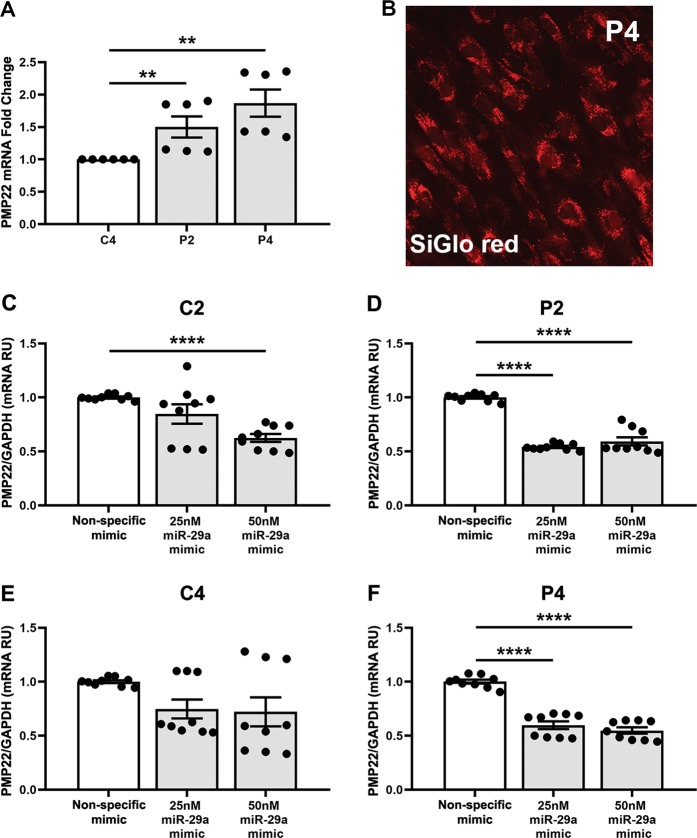
Correction of the human PMP22 transcript in dermal fibroblasts from CMT1A patients by miR-29a. **a** Elevated expression of PMP22 mRNA in cells from CMT1A patients (P2, P4), as compared with a control (C4) when treated with nonspecific mimic, and as shown by fold-change values. **b** Representative image of SiGlo red in transfected P4 dermal fibroblasts is shown. **c**–**f** Relative expression PMP22 mRNA levels after transient transfection of fibroblasts from age-matched control (C2, C4) and CMT1A patients (P2, P4) with nonspecific mimic, or 25 or 50 nM miR-29a mimics. Individual data points from *N* = 2–3 independent cultures per sample, with 2–3 technical replicates each, are shown. **p < 0.01, ****p < 0.0001, unpaired two-tailed Student’s *t*-test. RU, Relative Units

In order to demonstrate proof-of-concept that human PMP22 levels can be inhibited by miR-29a in cells from CMT1A patients, we extended our studies to primary dermal fibroblasts. We cultured skin fibroblasts from a CMT1A pedigree with confirmed PMP22 gene duplication obtained from the Coriell Institute [19]. Samples from nonneuropathic individuals with ages ranging from 21 to 57 years were used as age-matched controls. As expected, PMP22 mRNA levels are ~1.5-fold higher in CMT1A patient (P2 and P4) fibroblasts, as compared with cells from an unaffected control (C4) individual (Fig. 4a), when treated with nonspecific mimic. In order to determine if miR-29a can reduce elevated PMP22 levels in CMT1A patient fibroblasts, we performed transient transfections with miR-29a mimics (Fig. 4b–f). The miR-29a mimic was designed to be as physiologically relevant as possible while producing a measurable effect. To monitor transfection efficiency, we used a SiGlo red labeled siRNA, which reveals that at 48 h post transfection nearly 100% of cells are red (Fig. 4b). In addition, we confirmed that the CMT1A patient fibroblasts transfected with the nonspecific mimic still express 1.5–2.0-fold higher levels of PMP22 mRNA as compared with the normal individuals (data not shown). Significantly, PMP22 transcript abundance in CMT1A patient cells is reduced by 40–50% (****p < 0.001) after transient transfection with the miR-29a mimic (Fig. 4d, f). As indicated, the effect of miR-29a mimic repression is more robust in CMT1A patient cells (P2 and P4) than in cells from age-matched control individuals (C2 and C4) with normal PMP22 mRNA levels. We propose that this outcome is a reflection of the high abundance of miR-29a target sites in the affected samples.

**Fig. 5:**
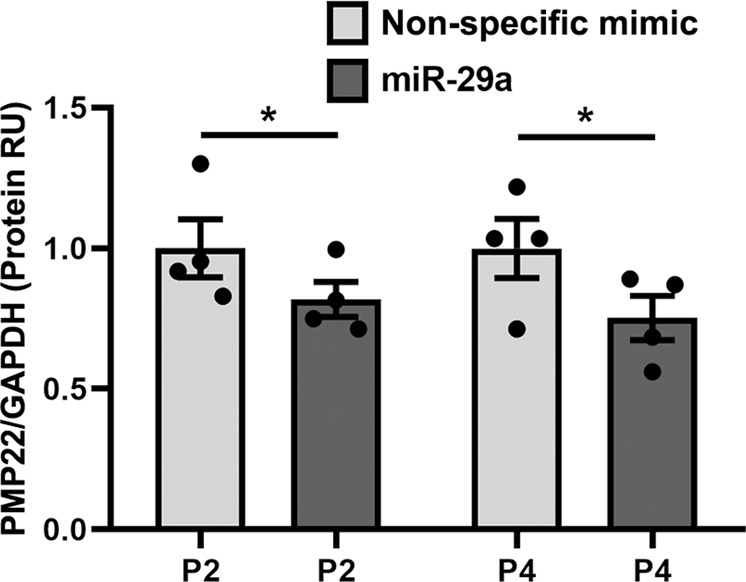
The levels of the human PMP22 protein in CMT1A patient fibroblasts are reduced after transfection with miR-29a mimics. Quantification of PMP22 protein in total lysates from independent cultures of CMT1A patient (P2 and P4) fibroblasts after transient transfection with nonspecific mimic, or with 50 nM miR-29a mimics. PNGase F digestion was used to facilitate the detection and the identification of the 18 KD PMP22 peptide. GAPDH served as the protein loading control. *N* = 3 biological transfection replicates, with 1–2 technical replicates each. *p < 0.05, paired one-tailed Student’s *t*-test. RU, Relative Units

Next, we tested if the reduction in PMP22 RNA was detectable at the protein level (Fig. 5). We performed quantitative western blots with anti-PMP22 and anti-GAPDH antibodies on whole cell lysates after transient transfection with miR-29a or nonspecific mimic. As shown in Fig. 5, the protein data from independent experiments with cells from two different CMT1A patients are in congruence with the mRNA results shown in Fig. 4, indicating that miR-29a contributes to the decrease in steady-state levels of PMP22. Together, these findings indicate that transient overexpression of miR-29a can lower PMP22 mRNA (Fig. 4) and protein (Fig. 5) levels in CMT1A patient fibroblasts by about 25–40%, which is within the desirable therapeutic window for this dosage-sensitive gene.

### MiR-29a restores mitotic activity of CMT1A dermal fibroblasts

**Fig. 6:**
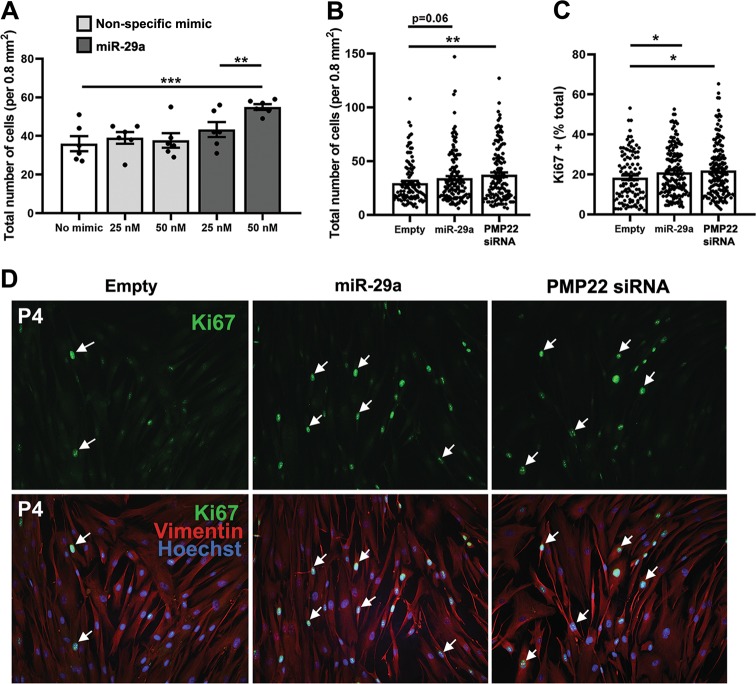
Transient transfection with miR-29a mimic or infection with AAV2-miR-29a promotes the proliferation of fibroblasts from a CMT1A patient. **a** Quantification of the total number of cells, from transiently transfected CMT1A patient 4 dermal fibroblasts, in randomly chosen fixed areas (0.8 mm^2^), after staining with Hoechst nuclear dye. **b** Quantification of the total number of cells, from empty, miR-29a, or PMP22 siRNA infected CMT1A patient two and four dermal fibroblasts, in randomly chosen fixed areas (0.8 mm^2^), after staining with Hoechst nuclear dye. **c** Quantification and combined analyses of Ki67+ cells from CMT1A patient fibroblast cultures indicate increased cell division after AAV2-mediated overexpression of miR-29a. **d** Representative images of CMT1A patient 4 infected with empty, miR-29a, or PMP22 siRNA AAV2 vectors, and stained with anti-Ki67 (green), intermediate filament protein vimentin (red), and Hoechst nuclear dye (blue). Images captured at X20 magnification. Data points from *N* = 2–6 independent cultures per patient are shown, with *N* = 6–170 areas each condition. *p < 0.05, **p < 0.01, ***p < 0.001, unpaired one-tailed Student’s *t*-test

In order to determine if miR-29a-mediated suppression of PMP22 had a functional consequence, we performed cell proliferation studies (Fig. 6). Dermal fibroblasts from CMT1A patients display reduced mitotic activity, which is the expected phenotype of elevated gas3/PMP22 protein [19, 29]. Indeed, upon transient transfection of the cultures with 50 nM miR-29a mimic, we observed increased proliferation of the cells. Quantification of total cell number from independent experiments using Hoechst nuclear dye revealed increased mitotic activity, as compared with nontransfected, or nonspecific mimic transfected cells (Fig. 6a).

To further study phenotypic correction upon ectopic miR-29a expression, we generated AAV2 constructs either expressing miR-29a or a siRNA-like hardened version of miR-29a. After 72 h of AAV2 infection with the indicated constructs, we stained the cells with anti-Ki67 antibodies, a marker of cell proliferation [30]. We used antivimentin antibodies, an intermediate filament marker, and Hoechst nuclear dye to label all cells. Quantification of total cell number (Fig. 6b) and Ki67-positive cells (Fig. 6c) from independent experiments indicates the number of mitotic cells in cultures from neuropathic persons increased significantly upon miR-29a overexpression and siRNA targeting of PMP22. This proliferative correction is evident in representative images from CMT1A patient fibroblasts upon miR-29a AAV2 infection (Fig. 6d). Notably, we observed the functional effect at or below ~30% AAV2 infection rate, which we quantified using a control reporter AAV2 (data not shown). Together, these results indicate that miR-29a is effective in ameliorating the cellular phenotype of PMP22 overexpression in dermal fibroblasts from CMT1A patients.

## Discussion

The studies described here provide proof-of-concept data for the efficacy of miR-29a in correcting the disease phenotype of cells from CMT1A patients. Our results provide some unique and promising features for further consideration in translational gene therapy efforts. In contrast to synthetic ASO therapeutics that would be delivered ubiquitously throughout the body, we are projecting the enhancement of endogenously expressed miRNAs in Schwann cells where they would be therapeutically beneficial by using AAV vectors. The conservation of the miR-29a gene regulatory mechanism among murine and human species allowed us to demonstrate the efficacy of miR-29a in affected cells from a neuropathic mouse model and in samples from CMT1A patients. By three different approaches, including synthetic mimics, transient transfection, and AAV2-mediated viral transduction, we show that miR-29a is able to effectively suppress steady-state PMP22 mRNA and protein levels, and this effect is beneficial in alleviating cellular disease phenotype. Significantly, the reduction in PMP22 is intermediate, which is the desired level for a dosage-sensitive gene. Recall, deletion of one or both copies of the PMP22 gene causes HNPP, an often painful, compression-induced peripheral neuropathy [31].

The dosage sensitivity of PMP22 dictates a careful approach to correcting the overexpression of the protein in neuropathic patients. As of now, there have been two clinical trials for CMT1A, both using orally available molecules. The first clinical trial was based on studies in mice using ascorbic acid to correct the expression of the overproduced PMP22 [32, 33]. While this multicenter clinical trial enrolled nearly 500 CMT1A patients over several years, ascorbic acid (vitamin C) supplementation did not provide benefits to affected individuals [34]. A recent international Phase 3 clinical trial for CMT1A used a pleiotropic drug therapy, and included a low-dose combination of baclofen, naltrexone, and d-sorbitol [35]. Formal publication on the results from this study has not been made public. In our lab, ongoing preclinical studies in animals use chaperone-inducing small molecules to alleviate the subcellular proteostatic stress caused by the overexpressed PMP22 gene [28]. Besides the mentioned small molecule drug therapies, in a recent study, PMP22 antisense oligonucleotides (ASOs) were utilized to treat C22 mice and CMT1A rats and reported a 50% reduction in the PMP22 mRNA, with significant improvements in myelinated axons and in nerve conduction velocity [11]. Each of these approaches used exogenous molecules to remedy the molecular and cellular phenotypes caused by PMP22 gene duplication and, due to the nature of the systemic applications, they could elicit undesirable off-target effects outside of the PNS upon extended administration. The miR-29a dependent strategy, in combination with a myelinated human Schwan cell targeting vehicle, would deliver the therapeutic molecule to the cells of interests, however such vector, including an AAV serotype, is yet to be identified. In addition, as myelinated peripheral nerves innervate the entire body and are protected by the blood–nerve barrier, the route of administration will have to be carefully optimized to assure efficacy, yet minimize potential off-target effects in other organs. One approach to overcome this latter obstacle will be to use a Schwann cell-specific promoter to drive the expression of miR-29a.

While miRNAs typically have multiple target genes, their cell-specific expression provides a unique endogenous regulatory mechanism for exploitation in gene therapy efforts. For example, miR-29a is expressed at high levels in oligodendrocytes of the CNS, in cells where the levels of PMP22 protein are below detection [36]. In comparison, miR-29a is low in Schwann cells, where it allows for PMP22 expression. Using bioinformatics and microarrays, we also identified miR-29a highly-expressed in actively proliferating Schwann cells, where PMP22 is low. Notably, the levels of miR-29a were decreased ~7-fold in differentiated, myelin forming Schwann cells [6]. By luciferase reporter assays and real-time RT-PCR experiments we confirmed an inverse relationship between miR-29a and PMP22 and demonstrated a reduction in PMP22 mRNA levels when miR-29a was high. On the other hand, inhibition of the endogenous miR-29a increased the steady-state levels of PMP22 mRNA and protein. The inverse functional relationship between miR-29a and PMP22 mRNAs was preserved in developing rat nerves, and in post-crush-injury adult mouse nerves [6]. The inverse relationship between PMP22 and miR-29a shown in our previous studies demonstrates the dynamic association between these two molecules. Identification of the specific seed sequence within the 3′UTR of PMP22 [6] and the conservation of this regulatory motif from rodents to human support the relevance of the miR pathway in governing PMP22 expression. In the current study, the luciferase reporter assays similarly indicate direct binding and translational repression of human PMP22 by miR-29a.

Since the discovery of miRNAs in the 1990s, the field has expanded from investigations on the role of miRNAs in regulating gene expression in specific cell types, to exploiting the pathway for disease therapeutics. For example, understanding the roles of miRs in regulating specific genes have led to new therapeutic targets in various benign and metastatic cancers [37]. Some miRNA-driven therapies have reached clinical trials, including a liposomal formulation of miR-34a in advanced hepatocellular carcinoma [38]. In an inverse approach, specifically inhibiting endogenous miRNAs have been explored for the treatment of hepatitis [39]. MiRs, including miR-29a, have also been identified as biomarkers of disease. For example, elevated expression of miR-29a, along with suppression of its target gene Robo1, may serve as a diagnostic or therapeutic target in gastric cancer [40]. Overexpression of miR-29a was also shown to inhibit cancer cell invasion in models of human retinoblastoma and lung adenocarcinoma [41]. In the CNS, miR-29a overexpression promoted apoptosis through the mitochondrial pathway, post intracranial aneurysm [42]. The diverse biological roles of miRs and their multiple putative targets present a major challenge in the design and delivery of microRNA-based therapies. However, not all of the potential binding sites identified in microRNA bioinformatics analyses are functional, necessitating a means to focus on validated targets. To address additional gene regulating effects of miR-29a, future RNA-seq studies will be able to identify the complete RNA targetome in Schwann cells via Argonaute (AGO) crosslinking. The field is working to improve mRNA target affinity and specificity to ameliorate side effects but for now, Schwann cell-specific delivery of miR-29a is the best option to prevent systemic effects and minimize toxicity.

The current study provides compelling evidence for the utility of miR-29a as a therapeutic approach for CMT1A, however, given the multiple gene targets of miR-29a, a suitable delivery vehicle must be identified prior to in vivo application. Here, we engineered an AAV2 particle expressing miR-29a, which in exploratory studies did not lead to efficient transduction of Schwann cells in mice (data not shown). However, in a published study, scAAV8-encoded miR-29a was successfully used to ameliorate hepatic fibrosis in mice [43]. In that study, a single intraperitoneal injection of the AAV8-miR particles successfully prevented and reversed CCl4-induced hepatic fibrosis, as assessed by biochemical and histological evidence. Therefore, with the development of a Schwann cell trophic AAV particle, it is conceivable that miR-29a will be an effective gene therapy agent for preventing and reversing nerve pathology due to PMP22 overproduction.
